# Clinical Proteomics for Solid Organ Tissues

**DOI:** 10.1016/j.mcpro.2023.100648

**Published:** 2023-09-19

**Authors:** William S. Phipps, Mark R. Kilgore, Jacob J. Kennedy, Jeffrey R. Whiteaker, Andrew N. Hoofnagle, Amanda G. Paulovich

**Affiliations:** 1 Department of Laboratory Medicine and Pathology, University of Washington School of Medicine, Seattle, Washington, USA; 2 Clinical Research Division, Fred Hutchinson Cancer Research Center, Seattle, Washington, USA; 3 Department of Medicine, University of Washington School of Medicine, Seattle, Washington, USA

**Keywords:** mass spectrometry, clinical proteomics, targeted proteomics, untargeted proteomics, tissue analysis, pathology, standardization, immunohistochemistry

## Abstract

The evaluation of biopsied solid organ tissue has long relied on visual examination using a microscope. Immunohistochemistry is critical in this process, labeling and detecting cell lineage markers and therapeutic targets. However, while the practice of immunohistochemistry has reshaped diagnostic pathology and facilitated improvements in cancer treatment, it has also been subject to pervasive challenges with respect to standardization and reproducibility. Efforts are ongoing to improve immunohistochemistry, but for some applications, the benefit of such initiatives could be impeded by its reliance on monospecific antibody-protein reagents and limited multiplexing capacity. This perspective surveys the relevant challenges facing traditional immunohistochemistry and describes how mass spectrometry, particularly liquid chromatography-tandem mass spectrometry, could help alleviate problems. In particular, targeted mass spectrometry assays could facilitate measurements of individual proteins or analyte panels, using internal standards for more robust quantification and improved interlaboratory reproducibility. Meanwhile, untargeted mass spectrometry, showcased to date clinically in the form of amyloid typing, is inherently multiplexed, facilitating the detection and crude quantification of 100s to 1000s of proteins in a single analysis. Further, data-independent acquisition has yet to be applied in clinical practice, but offers particular strengths that could appeal to clinical users. Finally, we discuss the guidance that is needed to facilitate broader utilization in clinical environments and achieve standardization.

## Highlights

• Immunohistochemistry workflows are poorly standardized.
• Mass spectrometry may complement existing tests for proteins in solid tissues.
• Targeted workflows could help clarify borderline immunohistochemistry cases.
• Untargeted assays provide relative quantification of 100s to 1000s of proteins.
• Guidance is needed to help clinical labs develop robust tools for solid tissues.

## Graphical Abstract

**Figure:**
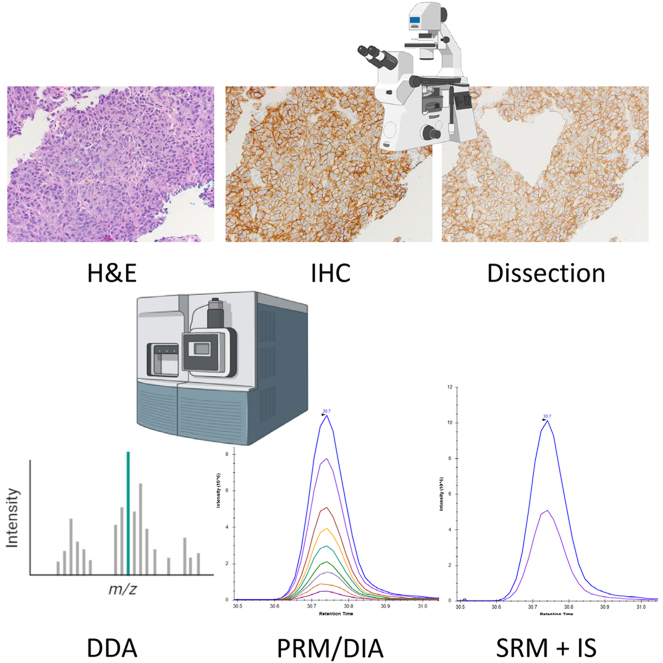

## In Brief

For many years, immunohistochemistry has been the primary tool in pathology for protein localization and quantification in solid tissues, but it is poorly standardized and suffers from practical limitations. The field is ripe for novel complementary technologies to help with diagnosis. This perspective focuses on the issues hampering immunohistochemistry and highlights how liquid chromatography-tandem mass spectrometry in particular is well-positioned to complement existing testing. We also discuss where guidance is needed to ensure rigor in clinical environments.

The examination of solid organ tissue is essential in the diagnosis, prognosis, and therapeutic management of cancer and other disorders. Components of this practice have existed for more than a century, including formalin fixation, paraffin embedding, and stains such as hematoxylin and eosin (1, 2, 3, 4). The introduction of immunohistochemistry (**IHC**) in the 1970s signaled a major shift in the field, specifically from diagnosis based primarily on morphology to a more comprehensive approach incorporating cell lineage and other protein expression information. Although IHC has broadly reshaped diagnostic criteria and advanced the practice of pathology, its rapid adoption was accompanied by a lack of standardization in workflows and reagents, hampering reproducibility (5, 6, 7, 8). Efforts are ongoing to improve standardization, particularly in the area of companion diagnostics, but these efforts may not address fundamental weaknesses of the technology, which would limit the long-term benefits of clinical tissue protein measurements in patient care. The field is thus ripe for introducing new complementary technologies for protein quantification in tissues that are able to overcome these issues, particularly for select applications. Techniques amenable to multiplexing are most desired, given the need for multianalyte panels to overcome the deficits of existing single biomarker tests in predicting responses to therapy. This *perspective* focuses on the challenges facing IHC and highlights how mass spectrometry, including liquid chromatography-tandem mass spectrometry (**LC-MS/MS**) in particular, could complement IHC to close gaps in existing testing using both targeted and untargeted acquisition approaches. We also discuss where further guidance is needed to ensure that rigorous assays are developed in clinical environments.

## Challenges in Immunohistochemistry Standardization

Immunohistochemistry utilizes anti-protein antibody reagents to bind and label antigens in tissue sections for visualization by microscopy. In the preanalytical phase, a sample is procured *via* biopsy or surgical procedure, preserved using formaldehyde in water (a.k.a., formalin; variable concentration, pH, time, and temperature), and embedded with paraffin wax to yield a formalin-fixed paraffin-embedded (**FFPE**) tissue block (9). In preparation for immunostaining, one or more sections (4–10 μm thick) of the block are cut using a microtome and mounted on a glass slide. Cut sections are subjected to deparaffinization, antigen retrieval (*e.g.*, with heat), and treatments (*e.g.*, biotin quenching) to prevent method-related artifacts. Finally, immunostaining itself consists of multiple incubation steps separated by rinsing: incubation with primary antibody against the target of interest, incubation with a reporter-linked secondary antibody conjugated typically *via* biotin to an enzyme (horseradish peroxidase), and addition of a chromogenic substrate (3,3′-diaminobenzidine) to produce color deposition at the site of antigen. Counterstaining is then performed to improve the visualization of labeled markers in relation to morphology. A pathologist views the results and renders an interpretation.

The introduction of IHC in the mid-1970s as ancillary testing to histologic staining represented a major step forward in reducing ongoing, well-documented inconsistencies in microscopy-based interpretation (10, 11, 12, 13, 14, 15, 16, 17, 18, 19, 20, 21, 22, 23, 24). However, despite the enormous and continually expanding benefit of IHC to patient care, its practice has struggled to meet the standards that we normally ascribe to clinical laboratory testing. As noted by Dr Clive Taylor (former Chair of Pathology at the University of Southern California and frequent contributor to this subject), the treatment of IHC has more closely mirrored that of the differential and special staining methods that preceded it in anatomic pathology (AP):

*“Not only is there much to be learned, but also there is much to be “unlearned”: the AP laboratory is handicapped by its legacy; it performs stains, not assays…”* (25)

The result is that IHC still exists today as more of an art than as a well-characterized and scientific assay (26). For example, judgments of quality in IHC are typically based on whether staining patterns please the eye, resulting in different laboratories differentially tweaking the practice of the same IHC test at different institutions (7). Proficiency testing (**PT**) results from external quality control organizations provide a window into the scale of irreproducibility that exists. Vyberg and Nielsen (27), for example, examined IHC performance encompassing more than 30,000 slides from 2003 to 2015 in the Nordic Immunhistochemical Quality Control international PT program and found that 20% to 30% of staining results in breast cancer and other diseases were "insufficient" for diagnostic use. Interlaboratory comparisons performed as part of clinical trials are equally sobering, showing discordance rates up to 75% when testing for treatment-related markers such as estrogen receptor (ER), progesterone receptor (PR), and human epidermal growth factor receptor 2 (HER2) (6).

Unfortunately, the measures of irreproducibility highlighted by PT and other published interlaboratory comparisons only tell part of the story, since such evaluations typically start with precut sections of the same FFPE block. More specifically, in addition to the effects of interlaboratory variability in immunohistochemical staining workflows, there exist inconsistencies in sample collection and processing (*i.e.*, the preanalytical phases of testing). In particular, the handling conditions for invasively-obtained solid organ biopsy samples can differ substantially from patient-to-patient in ways that laboratories cannot reasonably oversee or that may be challenging to document (*e.g.*, length of warm ischemia *versus* cold ischemia, (7)). The preanalytical variables in IHC are numerous, only partially investigated, and subject to conflicting findings regarding their effects on end results [reviewed in (28)]. Moreover, re-collection of a sample is typically impossible or at least highly impractical due to the invasiveness of collection (*e.g.*, tissue collected during surgery or endoscopy). This contrasts with the testing of many biofluids, where blood draw conditions can be more strictly defined and samples can often be recollected if there is a deviation from the procedure.

**Fig. 1:**
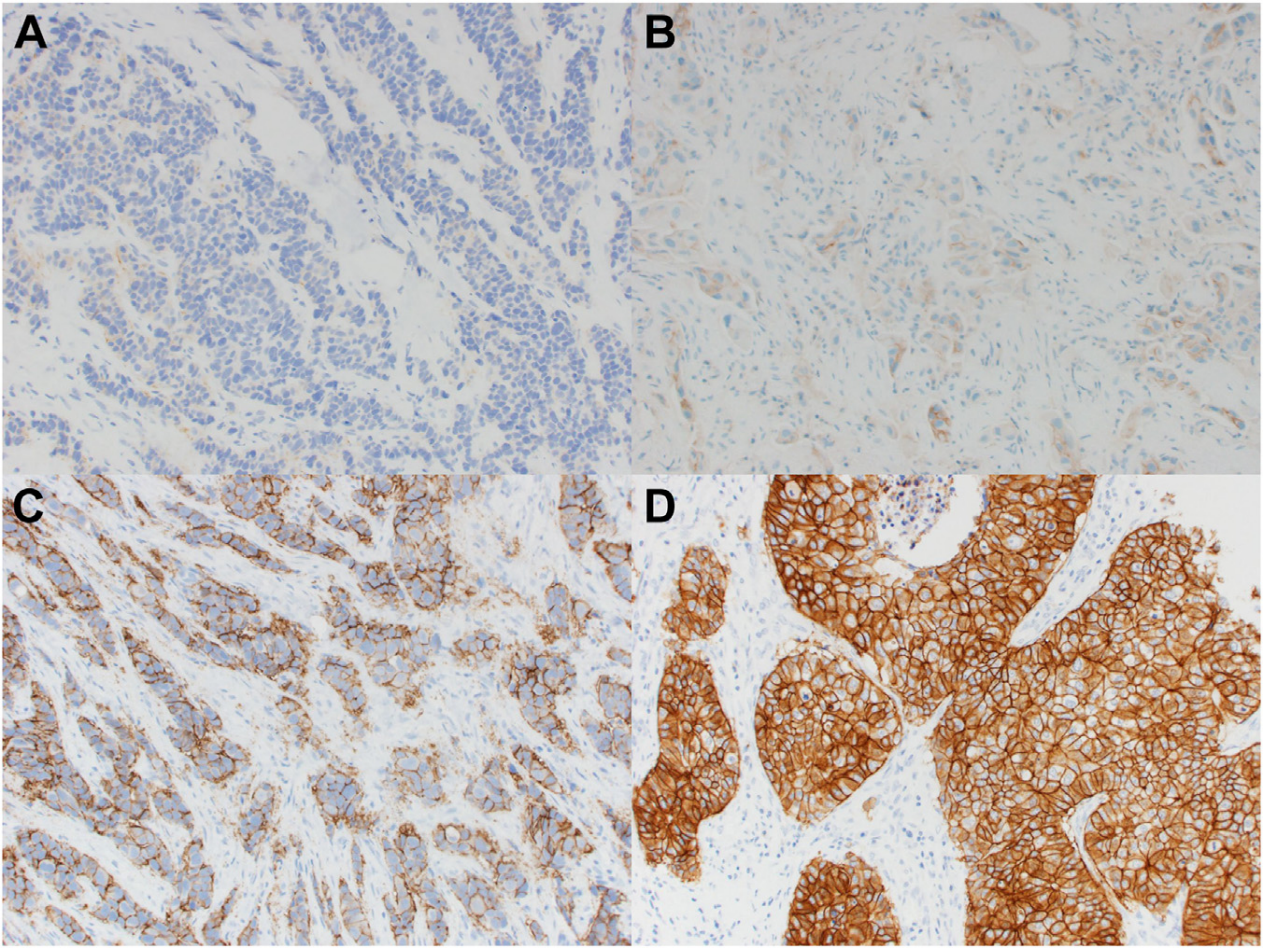
**Immunohistochemical (IHC) Interpretation for HER2 protein expression in Breast Cancer.***A*, IHC 0 (negative), no staining is observed or membrane staining that is incomplete and is faint/barely perceptible and in ≤10% of tumor cells. *B*, IHC 1+ (negative), incomplete membrane staining that is faint/barely perceptible and in >10% of tumor cells. *C*, IHC 2+ (equivocal), weak to moderate complete membrane staining observed in >10% of tumor cells. *D*, IHC 3+ (positive), circumferential membrane staining that is complete, intense, and in >10% of tumor cells.

The lack of standardization in IHC is particularly problematic for companion diagnostics, which are standalone tests used to assign patients to targeted cancer therapies. Such testing ultimately classifies patients into descriptive categories (*e.g.*, responder *versus* non-responder) but does so based on a semi-quantitative interpretation of staining patterns. Cases of breast cancer undergo separate IHC evaluations for HER2, ER, and PR, with the status of each interpreted based on the number of individual cells highlighted through the IHC reaction, typically with additional consideration of staining intensity. In the case of HER2, IHC results, on a scale of 0, 1+, 2+, or 3+ (Fig. 1), are paired with results from *in situ* hybridization (ISH) to determine the appropriateness of anti-HER2 therapy (29). More nuanced tiered scoring systems, such as the histochemical score (H-score), have been developed to account for tissue heterogeneity by using an estimate of the percentage of cells that are stained at different intensities (resulting in a weighted score from 0 to 300) (30). Although such developments capture increasingly granular information from tissue samples, the use of visually-driven metrics as proxies for the quantity of biomarkers is still problematic because quantification by the human eye is imprecise, and staining appearance will vary between laboratories. The situation is especially troublesome when evaluating samples with results near decision-point cutoffs (*e.g.*, HER2 1+ *versus* 2+), for which small differences in counting/scoring can affect categorization (7, 31). The effects can be dramatic and are at least partially responsible for the substantial variability in response rates to cancer immunotherapies observed in clinical trials (32, 33, 34, 35, 36). In one study, Lambein *et al.* (37) re-performed HER2 IHC evaluation for a set of 150 consecutive cases of invasive breast cancer that had been previously designated HER2-IHC-negative (0+ or 1+) based on IHC performed at a local laboratory. Overall concordance was modest at 86%. However, for cases previously scored as 0 at the local laboratory (n = 102), concordance was only 15%, with 78 of 102 cases recategorized as 1+ and 9 cases recategorized as 2+. In light of more recent clinical trials showing the treatment benefit of HER2-low cases with emerging therapies (*e.g.*, trastuzumab-deruxtecan), these differences could lead to real clinical consequences (38).

## Ongoing Efforts to Improve Testing

The potential for adverse effects from divergent practices in IHC was recognized by many early users. As the utilization and scope of IHC rapidly expanded, Dr Taylor pointed out the need to clearly and comprehensively define the steps and variables that could impact patient results. He and others have invoked a “total test approach to standardization” that encompasses the pre-analytical, analytical, and post-analytical phases of IHC (7, 8, 10, 25, 39, 40, 41, 42, 43, 44). However, the widespread alignment of practices with the total test philosophy has been slow, reflecting the scale and complexity of procedural factors as well as the practical challenges to modifying established IHC workflows. As of today, differences in practice between IHC users are likely to persist as a potential source of irreproducibility. However, as noted by Torlakovic *et al.*, (45) it is not necessarily desirable to fully align procedures, instruments, and reagents, and thus “freeze” IHC method development, as this would stifle innovation, limit the use of new products, and impose significant practical restrictions. Efforts to achieve standardization of results have instead focused on the development of tools to detect problems, both in the assay and specimen, as well as to achieve a more meaningful comparison of results between institutions. Initiatives on multiple fronts have been pursued, with several highlighted below.

One major pursuit is that of more systematically designed assay controls, carefully selected for each IHC application [reviewed in (45, 46)]. Historically, IHC controls have provided only a narrow window into gauging assay performance. For example, the use of archived tissue as an external positive control sample may demonstrate the adequacy of an IHC protocol for the detection of analyte at some amount or concentration, but that level is typically undefined. It may not be clear, for example, whether the IHC results are adequate for judging the presence of much lower or higher concentrations potentially under consideration in a patient sample. This ambiguity does not exist for most other qualitative assays found in clinical laboratories (*e.g.*, urine drug screening). In other such tests, a “positive” or “negative” result depends on the analyte concentration being measured above or below a specified numerical cutoff (with units composed of physical properties, *e.g.*, nanograms per milliliter). In particular, the lowest amount of a substance that can be reliably detected is typically identified as the limit of detection (LOD), a concentration at which test performance can be evaluated daily. As a step in this direction, an international committee of experts in IHC has suggested the deployment of so-called “immunohistochemistry critical assay performance controls” (iCAPCs) (45, 46). The iCAPCs are a set of tissues assigned to a particular IHC test (*e.g.*, appendix, liver, and tonsil assigned to pan-keratin). These tissues have well-characterized expression patterns for the antigen in question and, furthermore, express the antigen across a full range of diagnostically relevant levels. As such, typically at least one iCAPC contains cells or other structures with known low (but still “positive”) expression of the antigen, in turn providing a descriptive representation of an IHC assay’s LOD. Once test performance is established using iCAPCs (*i.e.*, primary external controls), secondary controls such as other archived tissue can be employed for more routine assay monitoring. The use of iCAPCs has been shown to reduce interobserver variability in IHC, for example, in BRAFV600 E testing (47).

For more quantitative IHC applications (*e.g.*, HER2), qualitative assay controls may be inadequate for assuring reproducibility, even if improved. To prevent errors in companion diagnostic testing, Dr Steven Bogen has instead emphasized the need to introduce traceable units of measure, international standards, and calibrators for quantitative assay monitoring (6). Proposals include on-slide calibration standards created *via* the deposition of a target protein (or its peptide epitope) in spots at known concentrations. One configuration enables IHC calibration traceable to an existing, widely available NIST standard (SRM 1932, a fluorescein solution). Calibration efforts have culminated with the recent announcement of the National Cancer Institute (NCI)-funded Consortium for Analytic Standardization in Immunohistochemistry, which should help enable laboratories to determine a quantitative LOD for companion diagnostic assays *via* a distributable set of well-characterized calibrators (5).

Many of the abovementioned efforts, including iCAPCs and slide-based calibrators, may help standardize the analytical phase of IHC, but they cannot assess sample quality. Additional strategies are needed to ensure that pre-analytical handling and processing are appropriate and have not compromised the immunoreactivity of the protein of interest in the sample (48)). Ideally, protein epitope integrity would be evaluated by using the same protein somewhere else in the tissue. However, the likelihood that the same protein is present at similar concentrations from sample to sample is very low. Further, the detection of the protein elsewhere in the tissue does not guarantee it is detectable in the area of interest. Rather than the protein of interest, it could also be possible to use other proteins that are present in relatively constant amounts in specific cell types. For example, more than 30 years ago, Battifora described vimentin as a general marker of sample processing quality (49), based on its ubiquity and vulnerability to fixation conditions. Others have since continued to advocate for the use of housekeeping or structural proteins to verify tissue quality. For example, Taylor has cited the potential of so-called quantitative internal reference standards, which consist of reference analytes in FFPE tissue (*e.g.*, desmin, caldesmon) for which the effects of fixation and antigen retrieval have been measured, quantitatively (using techniques other than IHC, perhaps at the individual cell level), and compared with those of key test analytes (7, 50). Once the properties of a reference analyte are established, it can be evaluated by IHC alongside the test analyte in patient samples (in a separate IHC reaction). Similarly, Neumeister *et al.* (48) described a tissue quality index, reliant on a panel of proteins with empirically defined relationships to delays in fixation (cold ischemic time) based on quantitative immunofluorescence studies. The authors demonstrated, for example, that measurements of cytokeratin, extracellular signal-regulated kinase 1/2 (ERK1/2), and phosphorylated heat shock protein 27 (pHSP-27) can be applied to breast to tissue to evaluate for adverse delays in processing that could result in false negative ER expression.

Besides the standardization of analytical and pre-analytical processes in IHC, calibrated image analyses could also serve to enhance reproducibility, particularly in the area of the interpretation of staining patterns [reviewed in (7, 30, 51, 52, 53, 54)]. The development of computer-based interpretation algorithms closely follows the trend to digitalize anatomic pathology workflows in general, which would facilitate remote pathology consultations, create permanent storage records, and help in teaching medical students and resident pathologists. Whole slide imaging, in particular, has provided a wealth of data for the development of artificial intelligence (AI) tools, including machine learning algorithms (52). With respect to diagnostic image analysis, computer-aided approaches improve accuracy *via* a reduction in visual and cognitive biases (30, 55). AI may become most impactful when it pushes visual interpretation beyond what might be considered reasonable for the human eye. For example, the evaluation of heterogeneity of proliferation markers in cancers is particularly challenging, but digital image analysis has, for example, improved diagnosis and prognosis when used to assess Ki67 expression in gastroenteropancreatic neuroendocrine neoplasms, compared to manual procedures (56).

## Is This the Best Path Forward?

The abovementioned strategies are encouraging for the future of IHC standardization, addressing concerns related to preanalytical, analytical, and post-analytical workflows. However, there are still a few issues that might prompt the development of complementary strategies in parallel, as the efforts described earlier continue to unfold.

One question to consider is whether there is really sufficient precedent to guarantee that a test like IHC could be broadly standardized. The belief that standardization of IHC is achievable partially hinges on the similarities between IHC and the enzyme-linked immunosorbent assay (ELISA) (7, 8, 57). Dr Taylor described ELISA as a quantitative “gold standard” for measuring proteins in serum and a model for IHC, noting:

*“[An] enhanced IHC assay would serve accurately to quantify analytes (proteins) in tissue sections, analogous to the use of the ELISA method in the clinical laboratory. ELISA employs similar principles and essentially the same reagents as are used in IHC but is subject to much more rigorous control at all levels”* (7).

Dr Bogen similarly points to the clinical laboratory and its aspirational models of performance, citing reported error rates of <1% in clinical chemistry, immunology, and hematology (6). However, it must be recognized that immunoassays are frequently *not* reproducible between manufacturers and assay platforms. This is true even for ELISAs that are FDA-approved/cleared and coupled with well-characterized reference calibration materials. An example of this phenomenon is the thyroglobulin immunoassay, for which traceability to reference material/calibrators has long existed and yet harmonization has never been achieved among commercial systems (58, 59). Further, while clinical immunoassays appear less prone to error in PT compared to IHC, this is partially due to the fact that only identical ELISA platforms are compared head-to-head in PT assessments (which were really designed to demonstrate that each clinical laboratory performs FDA-approved/cleared devices properly). Dr Mogens Vyberg highlights the inequity in juxtaposing error rates of IHC and another clinical testing in a 2019 editorial response to Bogen, writing that:

*“[Regarding differences in error rates]…the reasons are complex, because the above findings [for IHC] reflect not a single assay, but very many different assays for different analytes; even for the same analyte, different IHC laboratories may use 1 of 20 or more different antibodies in attempting to stain for, for example, keratin.”* (60)

Ultimately, both IHC and ELISA are techniques dependent on monospecific anti-protein antibodies, a factor that could prevent either from achieving harmonization. The remarkable failure of standardization of antibody reagents in research supports this assertion. These tools helped fuel a reproducibility crisis due to the challenges of cross-reactivity and batch-to-batch variability (61). While one might hope that antibody reagents somehow perform better in clinical environments, there are no specific reasons to believe this is the case.

Another question to consider is how limited multiplexing capacity may prevent IHC from achieving all of its potential. IHC is normally chromogenic and, in its standard form, can only be multiplexed x2 (*i.e.*, red and black). Performing additional analyses for each case in order to provide quality measures (*e.g.*, an internal quality control) can be laborious and tissue consumptive. Alternatively, IHC by fluorescence (*i.e.*, immunofluorescence) can be multiplexed more easily than conventional IHC, but the improvement is often modest and carries other practical disadvantages [reviewed in (62, 63, 64, 65)]. Commercial systems such as the Phenocycler (Akoya Biosciences) and other cyclic immunofluorescence approaches add many more channels (purportedly up 100 or more analytes) without the loss of spatial information, but they are expensive and require proprietary analyzers and reagents. The higher number of analytes is accompanied by time-consuming cycles of antibody staining, imaging, and fluorophore bleaching, potentially lasting days (66). Whether these new approaches could practically help assess tissue quality is not yet clear.

## Mass Spectrometry as a Complementary Technique

Because laboratories are faced with heterogeneous and constantly changing choices of antibodies for IHC, which could hinder reproducibility within and across testing sites, it may ultimately be sensible to move on from technologies that primarily rely on antibodies for protein detection. An ideal complement to IHC in clinical tissue protein detection would address the abovementioned concerns, enable the measurement of proteins *via* orthogonal principles, and better facilitate highly multiplexed analyses. Mass spectrometric methods might meet these needs. There are two general approaches to the analysis of solid tissues: (1) mass spectrometry imaging (MSI), which combines multiplexed protein detection with spatial information (analogous to multiplexed immunofluorescence) and (2) LC-MS, which can quantify many, many analytes from solubilized tissues. Both are potentially viable in clinical workflows using targeted or untargeted acquisition methods.

From the perspective of practicing surgical pathologists, the most obvious and intuitive applications of mass spectrometry to solid tissues involve imaging. The prototypical example uses matrix-assisted laser desorption/ionization (MALDI), which raster-scans a laser beam across the surface of a matrix-coated tissue section and ionizes the underlying biomolecules for spatially registered mass analysis (by TOF or FT-ICR) at 5 to 200 μm resolution (67, 68, 69, 70, 71). The identification of intact proteins from MALDI-MS is challenging, particularly for larger proteins that are not reliably fragmented for adequate sequence coverage (72). However, database searching options and approaches to protein identification *via* LC-MS/MS analyses performed in parallel are becoming more widely available (68, 72). MALDI imaging is not inherently quantitative, but several approaches exist to help make the workflow more quantitative [reviewed in (70)]. Importantly, the factors impacting quantification are complex and as a result, quantification by MALDI-MS lags behind LC-MS/MS-based approaches (73).

Due to the challenges inherent in intact protein identification by MALDI-MS (63), other imaging techniques have combined MS with the principles of IHC (64, 66, 74, 75, 76). Such techniques include mass cytometry imaging, which uses monoclonal antibodies conjugated to isotopically pure rare earth metal atoms in order to encode protein epitopes for simultaneous analysis of up to ∼40 markers at cellular resolution (77). Standard imaging mass cytometry subjects a sample to laser ablation, which then vaporizes and atomizes a small area of sample (less than 5 μm^2^). The resulting aerosol is introduced into an inductively coupled plasma (ICP) ion source and ions are detected by TOF mass analysis (66, 77). Similar or improved spatial resolution can be achieved using the principles of time-of-flight secondary ion mass spectrometry (TOF-SIMS), which uses a primary ion (*e.g.*, Ar^+^, O_2_^+^) beam for regiospecific sampling and generation of secondary ions from a test sample. Multiplexed ion beam imaging by time of flight (MIBI-TOF) is an implementation of TOF-SIMS, using the technology to image metal-tagged antibodies applied to tissue sections. In addition to rare earth metals, antibodies can also be labeled with organic molecules. For example, the Tag-Mass approach (78), a type of MALDI-IHC, uses a photocleavable peptide-based MALDI-MS method for targeted imaging of tissues. Yagnik *et al.* (63) developed a similar method that labels antibodies with both a fluorescent molecule (for standard immunofluorescence) and a photocleavable peptide for MALDI-MS detection. The dual labeling of targets allows standard immunofluorescence and MALDI-MSI on the same tissue sections.

The major appeal of MSI is the study of biomarkers in relation to intact tissue morphology. For example, imaging data could help determine surgical margins or determine the spatial distribution of immunomodulatory proteins in the tumor microenvironment, which could be beneficial (79, 80). Additionally, MSI methods could offer higher throughput compared to mass spectrometry techniques that are coupled to chromatographic separation. However, two drawbacks need to be recognized. First, at least some of the abovementioned MSI configurations do not actually break free from monospecific anti-protein antibody reagents and thus issues with reproducibility could still apply. Second, the capacity to achieve reasonable throughput for clinical environments is unclear. Mass cytometry, for example, requires an extended antibody incubation period (potentially overnight) and a relatively slow scanning process (MIBI-TOF scanning of a 100 μm × 100 μm field of view requires 3 min, which corresponds to 5 h for a 1 mm^2^ area) (76).

LC-MS/MS, the major conceptual alternative to MSI for tissue analyses, already has a significant presence in clinical laboratories for the analysis of biofluids, particularly in the areas of therapeutic drug monitoring, metabolic disease evaluation, endocrinology, and toxicology (81, 82, 83, 84, 85, 86, 87, 88, 89, 90, 91, 92, 93). Protein quantification by mass spectrometry has also taken hold (81, 94), owing to critical advantages with respect to specificity (95, 96, 97, 98, 99), sensitivity/dynamic range (96, 100, 101, 102, 103), overcoming assay interference (104, 105, 106), multiplexing (107, 108, 109), and throughput/turnaround time (103, 110). While some of these assays employ anti-*peptide* antibodies (for enhanced sensitivity through peptide immunoaffinity enrichment), the use of anti-protein reagents is generally avoided. Target quantification is based instead on the measurement of appropriately selected surrogate peptides that are readily generated by proteolysis. Multiplexed measurements are commonplace, a function of the instrumentation rather than the use of specialized reagents.

An important prerequisite to the successful transfer of LC-MS workflows to clinical care is compatibility with FFPE tissues. Fortunately, the use of LC-MS with solid tissue is already well established in the research space (when sampled in bulk from the whole section or from more intentionally selected areas using microdissection). In discovery proteomics, for example, LC-MS/MS analyses are routinely employed for comprehensive protein identification in FFPE tissues (see review by Gustafsson *et al.* (9)). A key finding in this body of work is that bottom-up proteolysis-dependent techniques, when coupled with heat-based protein extraction (heat-induced antigen retrieval (9, 111)), produce highly comparable results for FFPE and frozen tissue across 100s to 1000s of proteins at a time (112).

## Targeted LC-MS/MS Applications for Solid Tissue

**Fig. 2:**
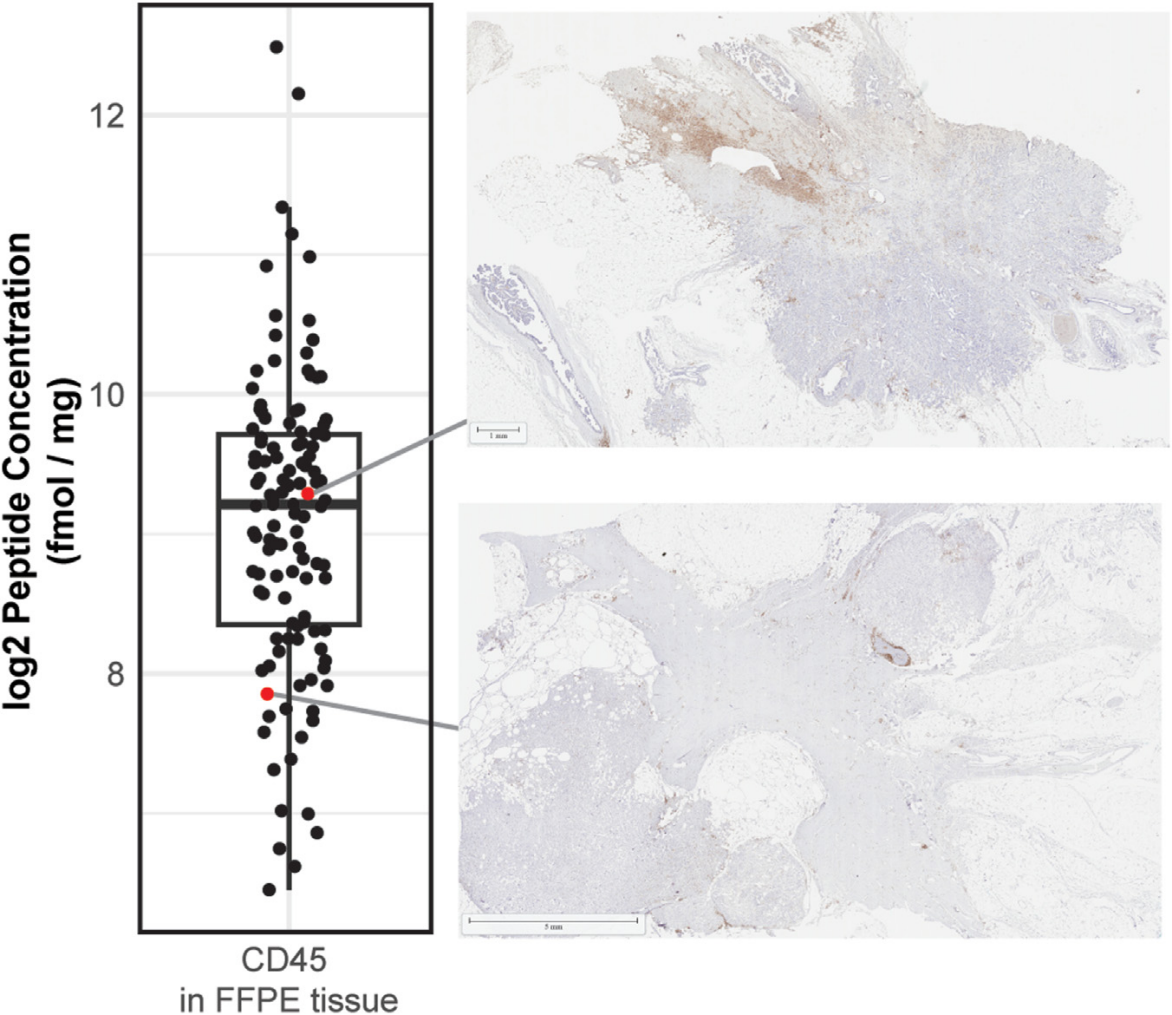
**MRM-MS associated with CD45 Immunohistochemistry.** MRM-MS results are shown at two different concentrations, demonstrating the correlation between visual IHC and a quantitative MRM-MS-based readout. Formalin-fixed paraffin-embedded tissues were stained with monoclonal mouse anti-human CD45 (Clone 2B11 + PD7/26, DAKO) and polyclonal anti-Rabbit IgG (Poly-HRP, Leica) before being counterstained with hematoxylin (Biocare).

The simplest translation of current clinical LC-MS/MS workflows to solid tissues would emulate the existing IHC practice of measuring a single protein to a few protein targets in each specimen. The use of multiple reaction monitoring (MRM) for this, in particular, could lend substantial objectivity and precision to this process (Fig. 2). Sprung *et al.* (113) demonstrated the feasibility of the approach, applying MRM to analyze over 100 peptides previously identified in frozen and FFPE renal carcinoma tissue using shotgun proteomic analyses. The results demonstrated equivalent precision (generally <20% CV) between measurements involving FFPE and frozen tissues, addressing the hypothesis that protein cross-linking in FFPE tissues would add undue variability in MRM measurements. Since this point, a growing number of publications have demonstrated the potential of LC-MS/MS to quantify the targets of cancer immunotherapy in applications that normally rely on IHC-based companion diagnostics (Table 1). A common finding among these reports is that more quantitative, objective analyses improve case stratification within and between diagnostic categories, more clearly delineating individuals likely to respond to immunotherapy or to harbor specific genetic alterations.

**Table 1:** Studies reporting application of targeted MRM-Based companion diagnostics (FFPE Tissue)

Reference	MRM targets (peptides)	IAE	FFPE tissues analyzed	Highlights
Hembrough 2012 (119)	EGFR (IPLENLQIIR)	No	NSCLC xenograft tumors (n = 10) NSCLC patient samples (n = 23)	• EGFR measurement normalized to total protein concentration • Results in amol per μg of total protein • LLOQ reported as 62 amol EGFR peptide loaded on column • Application to IHC+ NSCLC FFPE patient samples demonstrated EGFR levels over several orders of magnitude
Hembrough 2013 (129)	EGFR (IPLENLQIIR) HER2 (ELVSEFR) HER3 (LAEVPDLLEK) IGF-1R (GNLLINIR)	No	Breast cancer, 2 cohorts: 1) HER2 IHC variable (n = 10) 2) HER2 IHC 3+ (n = 19)	• Multiplexing facilitated identification of HER2 treatment resistance
Catenacci 2014 (118)	Met (TEFTTALQR)	No	Gastroesophageal cancer (n = 130) NSCLC	• MRM-based Met quantification reliably distinguished *MET* amplified tumors • IHC did not correlate well with MRM nor FISH gene copy number • Met >1500 amol/microgram was 100% sensitive and specific for *MET* amplification (based on ISH)
Steiner 2015 (120)	HER2, 6 peptides monitored	No	Breast cancer (n = 40)	• Six peptides monitored for HER2 produced highly correlated results • Five variables investigated as normalization factors^a^
Catenacci 2016 (117)	EGFR (IPLENLQIIR) FGFR2 (EAVTVAVK) HER2 (ELVSEFR) HER3 (LAEVPDLLEK) IGF-1R (GNLLINIR) Met (TEFTTALQR)	No	Gastroesophageal cancer (n = 139)	• “GEC”-plex assay • MRM-based measurement highly was concordant with *HER2/CEP17* ratio • MRM-based cutoff of 750 amol/microgram was highly specific for *HER2* amplification by ISH
Kennedy 2016 (128)	>500 peptide targets	Yes	Breast cancer (n = 3)	• Study utilized test battery approach to evaluate series of extraction and digestion procedure combinations • Metrics evaluated: protein recovery, digestion efficiency, sensitivity, precision, and repeatability
Nuciforo 2016 (114)	HER2 (ELVSEFR)	No	Breast cancer, invasive: HER2 0+ (n = 39) HER2 1+ (n = 49) HER2 2+ (n = 49) HER2 3+ (n = 133)	• Wide dynamic range observed in HER2 3+ group • HER2 >740 amol/microgram distinguished positive *versus* negative HER2 status by IHC/ISH standard testing (92% agreement, AUROC = 0.963). • HER2 >2200 amol/microgram associated with better survival^b^
An 2017 (36)	HER2 (ELVSEFR)	No	Gastric cancer (n = 237)	• 115-fold range of HER2 expression identified in HER2+ cases • HER2 >1825 amol/μg associated with improved survival^b^
Do 2020 (115)	HER2 (VLQGLPR) JAM1 (VTFLPTGITFK)	No	Breast cancer (n = 210)	• Evaluated five categories of normalization factors • JAM1 selected as preferred normalization factor for HER2 in place of extracted protein quantity • MRM-based HER2 measurement able to distinguish equivocal cases (HER2 2+/ISH negative *versus* 2+/ISH positive, *p* < 0.01)
Kennedy 2021 (116)	HER2 (GLQSLPTHDPSPLQR) +22 additional targets	Yes	Breast cancer (n = 119)	• Enhanced sensitivity achieved *via* IAE for HER2 measurement in FFPE and frozen tissues • Reported HER2 LLOQ 10 fmol/mg protein, using 20–50 μg of protein input • Multiplexed analyses for tumor compartment biomarkers corrected for tissue heterogeneity • Normalization by GAPDH levels improved concordance between immune-MRM-MS and predicate assays

Abbreviations: EGFR, epidermal growth factor receptor; HER2, human receptor tyrosine-protein kinase erbB-2; IAE, immunoaffinity enrichment; JAM1, Junctional adhesion molecule A; Met, hepatocyte growth factor receptor; NSCLC, non-small cell lung cancer.

a Percentage of tumor cells in the dissected area, surface area of dissected tissue, peptide concentration, total ion current in a separate full scan acquisition, and cytokeratin 19.

b In the setting of anti-HER2 therapy (traztuzumab).

The majority of studies in Table 1 focused on the quantification of HER2 in breast cancer. Nuciforo *et al.* (114) quantified HER2 in the set of 270 FFPE breast cancer samples that had been classified previously as HER2 0, 1+, 2+, or 3+ by IHC. As shown by these authors and in subsequent reports, HER2 quantity (measured in amol per microgram of total protein), generally correlates with IHC and amplification status by ISH, but there is a marked spread of concentrations with existing tiers and important differences with the IHC-based readout. For example, while a threshold of 740 amol/μg showed an agreement of >90% with IHC and ISH, a higher threshold (>2200 amol/μg) was associated with improvements in survival in patients treated with anti-HER2 adjuvant therapy. Do *et al.* (115) applied MRM-based HER2 measurement to 210 cases of invasive ductal carcinoma, investigating the capacity of quantitative HER2 results to differentiate the FISH status in HER2-equivocal cases (defined as IHC 2+, normally subject follow-up FISH testing). MRM-based HER2 quantification distinguished HER2+/FISH-negative from HER2+/FISH-positive cases with an AUROC value of >0.9, indicating that MRM-based measurement could be potentially utilized to avoid FISH in some cases. Kennedy *et al.* (116) applied HER2-MRM to 119 FFPE and 96 frozen breast cancer biopsy samples, highlighting the potential to identify HER2-low expression (IHC 1+ or 2) cases for which emerging targeting compounds (trastuzumab-deruxtecan) show benefit (34). In the study, using peptide-immunoaffinity enrichment (immuno-MRM), HER2 was quantified above a lower limit of quantification in all samples, improving the potential for stratification in this area.

Several studies have also focused on LC-MS/MS-based quantification of prognostic and therapy-selection markers in gastric and gastroesophageal cancers. As in breast cancer, a subset of patients who harbor HER2 amplification in gastric cancer (defined by IHC 3+ or positive FISH) are candidates for HER2-targeted therapy, based on a demonstrated benefit in the ToGA (Trastuzumab for Gastric Cancer) trial. However, substantial variations in response rates to anti-HER2 treatment have been observed in subsequent clinical trials, demanding a better approach to testing. Catenacci *et al.* (117) developed an MRM-based assay for quantification of HER2 in 139 cases, identifying a wide range of HER2 expression, even within specific groups. The degree of *HER2* amplification was linearly correlated with MRM-based HER2 measurement. Notably, the assay was multiplexed (building off a previous assay described in (118)), enabling simultaneous measurements of other oncoproteins, including Met tyrosine kinase receptor, epidermal growth factor receptor (EGFR), and receptor tyrosine-protein kinase erbB-3 (HER3), which was relevant in several specific patient vignettes, for which co-alterations in FGR2 or HER3 readily explained the treatment course observed in each case. An *et al.* (36) applied MRM-based HER2 measurement to a set of 237 FFPE cases of gastric cancer, identifying a 115-fold range of HER2 concentrations in HER2+ disease. A cutoff of 1825 amol/μg was determined to predict the benefit from the addition of trastuzumab to chemotherapy.

In addition to illustrating the potential therapeutic benefit of an MRM-based readout, these early studies have helped elucidate the steps of method development, which mirror those for assays in biofluids. For example, identification of surrogate peptides can begin with recombinant protein, proteolysis, and analysis with a high-resolution orbitrap MS (100, 119). The appropriateness of candidate peptides is characterized on the basis of sequence specificity and chromatographic features (*e.g.*, peak shape, retention time, precision of the LC-MS/MS analysis). The use of multiple peptides per target (*e.g.*, 6 peptides monitored for HER2 in (120)) is prudent early in method development to help ensure adequate operating characteristics of the final assay (100).

From here, method development may differ substantially from biofluid testing. One of the most important ways tissues may differ is in assay calibration. As we have learned for biofluids, accurate calibration for protein biomarkers is incredibly complex and is likely to be even more complicated for solid tissues (121, 122, 123, 124). As with measuring a protein in the blood, urine, or cerebral spinal fluid, reliable quantification in a tissue matrix will require the addition of stable isotope-labeled internal standards, which will be in the form of synthetic peptides, synthetic cleavable peptides (also called winged peptides), or intact proteins. These internal standards can help control for variation in sample preparation. However, the effectiveness of a spiked internal standard will depend on when it is added and how closely the conditions to which it is exposed match that of the analyte ultimately being tested. Unlike biofluids, to which internal standards can be added at initial processing, a generalizable solution for accomplishing this is not yet clear with solid tissues. Importantly, stable isotope-labeled internal standards can also be used as internal calibrators, which has been used for apolipoprotein A-I in human serum and is planned for use in reference measurement procedures being developed by the International Federation for Clinical Chemistry (125, 126).

In addition to using internal standards as internal calibrators to convert instrument signal to protein concentration, it is also possible to use external calibrators. For example, Hembrough *et al.* (119) described the creation of a standard curve by serial dilution of a light peptide from EGFR in a tryptic digest from the organism *Pyrococcus furiosus* that contained isotope-labeled peptide so that every serial dilution had the same concentration of labeled peptide. Catenacci *et al.* (118) described spiking increasing amounts of the light peptide into lysate from a formalin-fixed cell line. The use of these alternative matrices as the background for external calibrators borrows from clinical measurements in biofluids (*e.g.*, chicken serum as a calibration diluent for human serum thyroglobulin, (102, 127). More recent approaches have developed a reverse-curve approach to assay calibration, whereby the heavy isotope-labeled peptide is spiked into a matched matrix at different concentrations, keeping the unlabeled, endogenous peptide constant to serve as the internal standard (128). While this approach has the advantage of using a more favorable calibrator background matrix, this approach, and others before it rely on peptide calibrators instead of protein-based calibrators, which might be able to provide a higher order of test accuracy (122). Other approaches to external calibration could conceivably leverage slide-based calibrators, as is being explored for IHC, which would allow the calibration materials to be processed in parallel to patient samples, as is standard for current clinical LC-MS/MS assays of biofluids.

Tissue sampling and processing are two other analytical processes that will be quite different from biofluids and will greatly impact assay sensitivity and interpretation of results. Variations in sampling procedures could include whether to sample from a slide (or more than one) *versus* from the FFPE block. If sampling from a slide, it is possible to use laser capture microdissection (LCM) or manual microdissection. The amount of tissue, determined by the thickness of the section and the area sampled for slides and by the diameter of the punch and the depth of samples for tissue blocks, will also have a significant influence on the quality of the results. The majority of assays listed in Table 1 employed LCM of tissues on slides, which enabled some degree of control over tumor area (*e.g.*, 12 mm^2^ of a 10 μm thick section (117, 118, 119, 129)), whereas others used manual procedures (scalpel microdissection of 20 μm thick area delimited by an expert pathologist (120) or manual scraping of 3x 10 μm sections (128)).

The instrument signal or calculated concentration will need to be normalized to the amount of sample used in the digestion. Given differences in sampling practices as well as the variability in tissue content from one slide to the next, different normalization strategies have been also proposed (*e.g.*, quantity of analyte measured per total protein, per cells analyzed, per other protein biomarkers). As part of their study, Do *et al.* investigated five normalization factors, including tumor area (μm^2^), total cell count, total protein, total peptide, and light-heavy-peptide peak area ratios for 49 surrogate peptides from a range of epithelial cell-specific markers and common housekeeping proteins. From this last analysis, junctional adhesion molecule 1 (JAM1), an epithelial-specific protein was determined to be the best normalization factor (based on discrimination of HER2+/FISH-negative from HER2+/FISH-positive cases). As another example, Kennedy *et al.* (116), described the measurement of HER2 as a part of a 23-plex immuno-MRM-MS assay. In that study, normalization of HER2 levels to glyceraldehyde 3-phosphate dehydrogenase (GAPDH) improved concordance between immuno-MRM-MS and predicate assays for tumor classification. It is important to note that while the described normalization factors were beneficial in these individual studies, the potential limitations of each will require further consideration to not lead to misinterpretation. For example, GAPDH has been utilized as a normalization factor for gene or protein expression in other contexts (130, 131), but it is not perfect and could, for example, be affected by the variability in glycolytic capacity between tumors. Further studies (ideally larger and prospective) correlating normalized results and clinical outcomes could help clarify these issues.

After the tissue has been sampled, it must be processed and digested prior to analysis by LC-MS/MS, which must also be optimized. Kennedy *et al.* (128) evaluated many different combinations of protein extraction and trypsin digestion methods and identified an approach that yielded high precision across many analytes as well as excellent correlation between paired FFPE and frozen tissues.

For low-abundance proteins, it may be necessary to utilize peptide immunoaffinity enrichment to achieve better sensitivity. For clinical care, it has been difficult to measure low-abundance proteins directly in biofluids, especially using the normal to high-flow conditions that are most commonly available in those laboratories (200–1000 μl/min). Instead, peptide immunoaffinity enrichment can enhance sensitivity, using anti-peptide antibodies immobilized to a solid support (*e.g.*, magnetic beads) to selectively isolate and enrich specific peptides from complex biological samples. This approach has been greatly beneficial, as has been shown for the high-sensitivity quantification of serum thyroglobulin (102, 127) and in other applications (see review by Neubert, *et al.* (94)). As noted earlier, several tissue-based assays have included peptide immunoaffinity enrichment, including a highly multiplexed analysis of breast cancer tissue (116).

## Untargeted LC-MS/MS Analyses for Solid Tissues: the Example of Amyloid Typing

Although targeted analyses are most similar to the existing application of IHC, the use of untargeted analyses should also be considered. In particular, the translation of high multiplexing capacity directly to medical care is highly appealing, given the tremendous gain over IHC in deciphering the breadth of phenotypic data contained in biological samples. A potential hindrance to clinical application is that the high sensitivity and specificity of modern untargeted LC-MS/MS workflows (and, in turn, their reliability) are imparted by instrumentation atypical of clinical laboratories, namely, nanoflow chromatographs and high-resolution mass analyzers (*e.g.*, orbitrap). However, the plausibility of leveraging these systems clinically is no longer in question, given the success of clinical LC-MS/MS-based amyloid typing, which generally employs sub-microliter-per-minute flow rates and data-dependent acquisition (**DDA**) run on hybrid-orbitrap mass spectrometers (132, 133, 134). Furthermore, due to continual improvements in mass spectrometry instrumentation, highly sensitive, comprehensive protein analyses are increasingly possible at higher flow rates (135, 136), which will make running these tests more robust for new users.

The chemical typing of amyloid exemplifies the characteristics of applications for which untargeted LC-MS/MS analyses may be well suited. Systemic amyloidosis is a heterogeneous group of conditions in which proteins prone to misfolding deposit in tissues and result in organ damage. The correct identification of protein contained in amyloid deposits is essential for guiding treatment. However, more than 30 endogenous proteins and exogenous substances (*e.g.*, enfuvirtide) can contribute to amyloid formation, posing an analytical challenge (137, 138, 139, 140). Even if well-performing reagent antibodies existed against all amyloidogenic proteins, an immunohistochemical evaluation for each would exhaust all available tissue in many situations and require significant time for interpretation. Meanwhile, untargeted LC-MS/MS analyses do not require additional tissue consumption when considering additional amyloid type-specific proteins. Furthermore, a broadened proteomic examination allows for the detection of amyloidogenic substances that may not have otherwise been considered (or previously known), as well as additional markers useful for gauging the adequacy of amyloid sampling (*i.e.*, the amyloid-*associated* proteins: serum amyloid P, apolipoprotein E, victronectin, apolipoprotein A4, and clusterin (141)). Of further benefit to amyloid typing specifically is that the database searching process itself does not require peptides generated from proteolytic digestion to be consistent from sample-to-sample, which is critical, since the proteins found in amyloid deposits appear truncated in unexpected ways (142).

Other similarly imposing applications to subtype tissues exist in histopathology, requiring the consideration of growing lists of protein markers for which suitable anti-protein antibody reagents may not be available. One example is the subtyping of pituitary adenomas, traditionally distinguished by pituitary hormone expression—that is, growth hormone, prolactin, adrenocorticotrophic hormone, thyroid stimulating hormone, luteinizing hormone, and follicle-stimulating hormone. In addition to these proteins, the evaluation of transcription factors (*e.g.*, PIT-1, SF-1, T-PIT) is needed to meet the standard of care (143). In general, higher multiplexing will become increasingly important in medicine, particularly considering that a single or a handful of biomarkers are often insufficient to stratify patients in treatment categories (144). Further, multi-arm precision oncology trials require an assessment for the expression of many proteins, and IHC is a bottleneck.

To summarize these factors more generally, untargeted LC-MS/MS analyses provide benefits in applications for which: many proteins must be examined, there is value in a broader examination of proteomic features, and/or proteins may be truncated or otherwise modified post-translationally in ways we cannot predict. In future clinical practice, solid tissue applications in which untargeted LC-MS/MS methods are useful will be determined by these factors, as well as by the ease with which a targeted method could eventually be developed. For example, certain situations that are analogous in some respects to amyloid typing may not suffer from the effects of protein truncation and thus be more amenable to the identification of surrogate peptides for the proteins of interest. Meanwhile, robust and broadly encompassing proteomic data could prove especially valuable in practice when dealing with other ongoing sophisticated challenges in anatomic pathology, particularly if effectively integrated with genomic information (145).

## The Appeal of Data-Independent Acquisition in Clinical Testing

As untargeted LC-MS/MS analyses see additional clinical applications, one question to consider is whether DDA is the best we can do. In DDA, the instrument acquires fragmentation spectra for peptide precursors that were detected in wide mass-range “survey” scans (146). While the approach is capable of detecting thousands of proteins, it has several drawbacks. One is that an under-sampling of peptides occurs when evaluating complex mixtures (147), since the number of eluting peptides will exceed, by definition, the number of precursor ions for which fragmentation spectra can be acquired (148, 149). Furthermore, differences in chromatography from injection-to-injection influence which peptides are selected for fragmentation, resulting in poor reproducibility in identifications at the peptide level, even across sequential injections of the same sample. Meanwhile, since the highest abundance precursor ions are selected for analysis, low abundance ions are variably ignored, leading to reduced sensitivity of the analysis, especially for low abundance proteins. In one study, Tabb and colleagues (149) analyzed 3 sample mixtures in 144 LC-MS/MS experiments performed across eight instruments (Thermo LTQ and Orbitrap) to demonstrate that peptide detection across pairs of technical replicates only overlapped by 35% to 60%. Protein detection was more reliable, but still underwhelming (70–80% overlap). Quantification also suffers, in part due to a reliance on the integration of MS1 chromatographic peak areas or spectral counting (150). Due to chromatographic interference from overlapping peptides with the same precursor ion *m/z*, the former approach is typically not a viable option for the quantification of peptide content in complex mixtures. In contrast, a spectral count is the total number of spectra identified for a protein, which can correlate more reliably with protein abundance in some complicated sample types (151, 152). The measure has undergone multiple iterations to account for specific challenges, as reviewed elsewhere (151, 152). For example, one form used in amyloid typing is the normalized spectral abundance factor (NSAF) (141). The NSAF takes into account the conundrum that longer proteins will produce more detectable peptides (potentially conflating the quantitative interpretation). To facilitate comparison between samples, the measure is further normalized by all spectral counts within a sample per the following formula (153):

$$NSAF_{k}=\frac{{\left(SpC/L\right)}_{k}}{\sum_{i=1}^{N}{\left(SpC/L\right)}_{i}}$$

where NSAF_k_ is the NSAF for a specific protein with SpC spectral counts and L length, and N is the total number of proteins identified in the sample. Despite iterative improvements to the spectral counting principle, comparing SCs between two proteins may not accurately reflect their relative abundances in some cases (a topic beyond the scope of this perspective) (152). This may not be problematic for applications focused on the identification of the most abundant (or nearly most abundant) protein in a sample (*e.g.*, amyloid typing), but could prove limiting in other applications, particularly if greater precision is required or the quantity of a low abundance target is the goal of the assay.

The alternative untargeted acquisition method to DDA is data-independent acquisition (DIA). DIA, like DDA, employs a full *m/z* range survey scan, but in place of computer-driven precursor ion selection for MS/MS, the instrument accumulates all precursor ions for fragmentation across *fixed* sets of *m/z* isolation windows that, together, span the entire *m/z* range. Fragmentation spectra are mixed as a result (and therefore of higher complexity), but now contain data corresponding to all eluting peptides. Eliminating the stochastic precursor ion selection biased toward higher abundance peptides can improve consistency in the analysis and facilitate data capture for low abundance targets. Multiple studies suggest the approach improves consistency in protein/peptide identifications as well as quantitative reproducibility (150).

Studies of DIA applied to solid organ tissues have highlighted the benefits of the approach [Table 2, (4, 154, 155, 156, 157, 158, 159, 160, 161)]. For example, Hou and colleagues used DIA to identify putative biomarkers in esophageal carcinoma (155). In that study, 116 of 120 differentially regulated protein biomarkers identified by DIA were confirmed by targeted multiple reaction monitoring-mass spectrometry (MRM), indicating that quantification by DIA can come close to what is considered the current gold standard for mass spectrometry-based targeted protein quantification (162). Additionally, the authors were able to leverage the peptide transition information from the DIA experiments in the development of their MRM method for verification studies. In other studies by Weke (157) and Keme (158), the application of DIA identified many more proteins compared to previous studies that applied DDA to similar tissues.

**Table 2:** Reported applications of DIA to solid tissues in cancer proteomics

Reference	Solid tissues evaluated^a^	Protein analysis results	Highlight(s)
Hou 2015 (155)	Paired tissues: Esophageal squamous cell carcinoma tissue and adjacent normal tissue (n = 10, frozen tissues only)	1758 proteins quantified; 467 proteins differentially expressed	High correlation between quantitative information derived from DIA (SWATH) and co-performed MRM experiments
Gao 2017 (160)	Paired tissues: Hepatocellular carcinoma (HBV-associated) and adjacent normal liver tissue (discovery set n = 14, verification set n = 6, frozen tissues only)	4216 unique proteins quantified; 338 differentially expressed	Aspects of metabolic reprogramming identified; DIA (SWATH) data consistent with previous western-blot/IHC-validated results
Schwarzfischer 2017 (156)	Burkitt lymphoma: 5 frozen, 5 FFPE DLBCL: 6 frozen, 9 FFPE	Frozen: 2938 proteins quantified, 1125 differentially regulated FFPE: 1442 proteins quantified, 404 differentially regulated	Quantitative results reproducible between frozen and FFPE tissues; corroborating metabolomic studies were performed, demonstrating high secretion of pyruvic acid in BL compared to DLBCL
Keam 2018 (158)	Paired tissues: Prostate cancer (PCa), before and 14 days after radiation therapy (n = 8 patietns, comprising 2 with frozen and FFPE tissue, 1 frozen only, 5 FFPE only)	>5000 proteins quantified, 49 differentially expressed	Deeper proteomic analyses obtained for prostatic tissue by DIA compared to previously reported MS pipeline applied to PCa (159)
Zhu 2019 (4)	Multiple prostate cancer cohorts (frozen and FFPE tissues); multiple diffuse large B-cell lymphoma cohorts (n = 41 patients in first cohort, n = 52 patients in second cohort, 113 FFPE samples)	3030 proteins quantified in one PCa cohort, 4144 proteins in second cohort	Panel of protein biomarker candidates identified for PCa diagnosis; myeloperoxidase identified as prognostic marker in DLBCL
Janacova 2020 (161)	Endometrial cancer (n = 34, all FFPE): 15 – previous tamoxifen treatment (ET) 19 – no previous tamoxifen (EN) 11 – adjacent myometrium	904 proteins quantified; Differential expression of calcyphosin (CAPS) and stathmin confirmed (STMN1)	CAPS and STMN1 also appeared useful as prognostic indicators
Marchione 2020 (159)	18 tissue types evaluated using HYPERsol protocol + separate analyses of 32 archival samples of malignant peripheral nerve sheath tumor (MPNST), melanoma, and synovial sarcoma	4000–5000 proteins/sample	DIA-based analyses applied to distinguish histomorphologic mimics
Weke 2022 (157)	Glioblastoma (GBM) FFPE microdissections (n = 5)	>1700 proteins identified; >1400 quantified	Leveraged multiple sample processing approaches; Protein identifications increased compared to DDA; High quantitative precision for GBM markers: GFAP, FN1, VIM, and MBP

Abbreviations: BL, Burkitt’s lymphoma; DLBCL, diffuse large B-cell lymphoma; HYPERSol, high-yield protein extraction and recovery by direct solubilization; MPNST, malignant peripheral nerve sheath tumor; MRM, multiple reaction monitoring; PCa, prostate cancer; SWATH, sequential window acquisition of all theoretical spectral.

High-yield protein extraction and recovery by direct solubilization.

a Samples other than solid tissues are excluded from this table.

Whether these reported advantages of DIA in research will directly benefit patient testing remains to be seen. Furthermore, the notion of better reproducibility of DIA is not without debate or potential caveats (163), and the specific limitations of DIA should be considered. These include higher complexity of the data processing since spectra are mixed and must be deconvoluted. DIA may also be a lower throughput than DDA since the generation of spectral libraries may be needed (154). Effective use of this technology will require successful collaboration between end users in clinical laboratories and computer scientists. For example, an understanding of false discovery rates is beyond the scope of most practicing pathologists. Nonetheless, better reproducibility at the peptide level, if achievable, is appealing clinically for several reasons. Raw data that are more amenable to replication across injections can better facilitate the development of robust quality assurance measures mirroring those already in practice for targeted methods run on triple quadrupole mass spectrometers (*e.g.*, transition ion ratios). Better reproducibility at the peptide level [*e.g.*, >90% overlap in peptide lists between technical replicates (154)] should also benefit the identification of mutations or polymorphisms in patient samples wherein data corresponding to specified peptides will not be missing. Finally, more comprehensive and reproducible data will be more amenable to reexamination as new biomarkers are discovered. Also notable is that open-source options are becoming more prevalent for data processing and should be eventually adaptable into pipelines appropriate for use in clinical environments, given that this has already been demonstrated when handling shotgun proteomics data [*e.g.*, Crux Pipeline (141)].

## Guidance is Needed for Reliability and Standardization

The limited clinical application of mass spectrometry in solid tissues to date reflects a number of factors, including potential costs but also a range of requirements specific to the use of the technology in patient care. Many prerequisites, including those relating to test quality, are defined in government regulations, whereas others may be institution-specific. In the United States, the Centers for Medicare and Medicaid Services regulates all clinical laboratory testing, *via* the Clinical Laboratory Improvements Amendments of 1988 (CLIA). The specific rules governing clinical laboratories can be found in Code of Federal Regulations, Title 42, part 493 (42 CFR 493, “Laboratory Requirements”). While many of these regulations have general implications for operating a clinical lab, successful adherence to others (*e.g.*, proficiency testing, method performance verification, and quality control) must be accounted for on an assay-by-assay basis.

Key areas that must be addressed include method validation and performance verification. Generally speaking, assays using mass spectrometry-based platforms are considered *high-complexity* and, with few exceptions, are not FDA-cleared or approved. In addition, almost all mass spectrometry-based assays are categorized as *laboratory developed tests* (LDTs), meaning *in vitro* diagnostic tests that are designed, manufactured, and used within a single laboratory. As a result, despite the overall improvements in analytical sensitivity and specificity, these tests still have the potential for variability between laboratories. The lack of regulatory clearance or approval means that these tests are subject to additional requirements during method validation and verification of performance characteristics. According to regulation § 493.1253, developers of such assays must (at a minimum) establish assay accuracy, precision, analytical specificity, reportable range, and reference intervals. *How* these performance characteristics are established or judged is not detailed by government regulations, and thus is still subject to the discretion of a laboratory medical director. Accreditation organizations, such as the College of American Pathologists (CAP), set certain baseline standards. For example, CAP checklists include brief specifications of an evaluation of test accuracy and the suitability of samples used during verification. However, this guidance is limited and recommendations for method development and validation must be found elsewhere.

One source of guidance in test design and validation is the Clinical and Laboratory Standards Institute (CLSI), a non-profit organization serving device manufacturers and those clinical laboratories that are developing and running LDTs. CLSI creates standards of practice across areas of laboratory medicine based on expert consensus, with the intent of improving testing quality, safety, and efficiency. The standards cover many aspects of method development, validation, performance verification, and post-implementation monitoring. Some guidelines address mass spectrometry, but are not all-encompassing, and do not specifically address analyses involving solid tissues. These guidelines include, C50 (“Mass Spectrometry in the Clinical Laboratory: General Principles”), C62 (“Liquid Chromatography-Mass Spectrometry Methods”, recently updated in 2022), and C64 (“Quantitative Measurement of Proteins and Peptides by Mass Spectrometry”). As detailed by Lynch in 2016 (164), the impetus for development of these documents is the widening user-base and a need for standardization, as well as to fill in gaps not addressed by other guidance documents (*e.g.*, the FDA’s Guidance for industry: Bioanalytical method validation). Document C62, in particular, offers guidance with respect to LC-MS methods, focused on quantitative analyses performed on tandem mass spectrometers (with a special emphasis on triple quadrupole mass spectrometers). Assay elements included in this guidance document include calibration, validation, quality assurance, quality control, among others. Document C64 built upon C62, focused instead on the quantitative measurement of proteins, recognizing that protein and peptide analyses involve unique challenges and additional workflows. One other key recognition in the document is that although some proteins may exist as a single specified molecular composition, most will exist as a complex family of related proteoforms, differing in length, amino acid sequence, and post-translational modifications. As acknowledged in C64, this reality may stifle standardization and harmonization since two assays (just like different immunoassays) may not actually measure the exact same target or set of targets, making the selection of the analyte (*i.e.*, proteotypic peptide) an important step.

The transition to measuring proteins in tissues represents another step forward where consensus and guidance will be needed. Of course, many of the challenges affecting IHC will undoubtedly also need to be addressed for mass spectrometry-based approaches. Basic questions to consider include:

• What is an appropriate form of calibration?
• Can the appropriate matrix be used for analytical validation?
• What are suitable quality control materials?
• What are the most appropriate (or minimum) validation studies?
• How should proficiency testing be performed?
• What are appropriate quality assurance measures?

There is some precedent for answering these questions: (1) Existing amyloid typing pipelines and (2) targeted quantitative assays developed in CLIA-regulated environments. With respect to untargeted analyses, in their 2013 perspective, Theis and colleagues (142) defined what steps in the workflow could potentially contribute to variability in the processing of samples for amyloid typing. Unsurprisingly, many of the factors that they identified overlap with those that affect immunohistochemistry. *E.g.*, just as with IHC, LC-MS/MS could be affected by specimen processing features (*e.g.*, fixation technique), sample preparation factors (*e.g.*, antigen retrieval), and tumor heterogeneity. Other steps are specific to LC-MS/MS: choice/setup of collection technique (*e.g.*, LCM), chromatography, etc. To meet CLIA requirements, Theis describes addressing the six criteria required for all LDTs: Accuracy, analytical precision, analytical sensitivity, analytical specificity, reference range, and reportable range. However, it is clear that the experiments performed to address the six criteria will not be the same for all tissue assays. In addition, the interpretation of untargeted measurements aimed at tissue subtyping is very different from targeted assays aimed at quantifying a small number of proteins. Further, while one benefit of untargeted proteomics is the ability to discover new amyloidogenic proteins, it is less clear how confirmation of those discoveries should be performed in a CLIA laboratory, particularly in the absence of well-characterized standards (including for amyloid). The field would greatly benefit from more general consensus on what experiments should be performed to validate untargeted LC-MS/MS assays in tissues, although the previous examples in the literature may be a helpful starting point to answer the questions listed above (140, 141, 142).

Since targeted quantitative LC-MS/MS assays in tissues are more analogous to what is normally done in clinical laboratories, standard approaches to validation may be more appropriate with less ambiguous interpretation and subjectivity. Most targeted assays described to date have included some form of validation, although there are many elements of robustness that still need to be assessed. Method comparisons have generally been performed against IHC (or other traditional immunoassays), putting laboratories in the position of asserting assay acceptability compared with a potentially inferior gold-standard technology. Effects of tissue heterogeneity are likely to be an ongoing area of study; normalization will be particularly important in this regard. As described above, multiple strategies have been applied to the normalization of HER2 in tissue samples. While some of these approaches produce encouraging findings in early studies, the peculiarities of each and the potential for misinterpretation will have to be investigated. The challenge of normalization is already a major problem in proteomics more generally and is described elsewhere (131, 165).

## Where Do We Go From Here?

At present, although MS may be a viable alternative to IHC in many respects, it still suffers from significant concerns, many of which are relevant to IHC. This raises the question of, “how do we move forward?” We envision 3 steps in the development of best practices in clinical tissue mass spectrometry.

### Development of Programs to Share Clinical Cases Between Institutions

As noted earlier, tissue analyses almost universally lack standard materials. Furthermore, for LC-MS/MS analyses of proteins in general, there are typically no relevant PT materials available. This leaves laboratories in the precarious position of trying to verify the quality of their own assays. The latter might include retesting previous cases, which can demonstrate consistent performance over time, but does not speak to the accuracy of the analytical method or the strategies used in data processing (*i.e.*, it is possible to be wrong *twice* if nothing about an assay has changed). For quantitative assays, we might borrow from alternative assessment programs for LC-MS/MS assays of proteins in biofluids, which were designed to help promote harmonization. For example, clinical laboratories share samples biannually to assess the comparability of thyroglobulin testing across laboratories (127). The multiplexed nature of tissue MS results will make similar programs particularly important, since interpretation is more complicated.

### Improve Raw Data Accessibility

Development of best practices in data analysis and interpretation will require data sharing in order to directly compare data-processing pipelines. Data sharing has become increasingly robust in the research space, particularly through the efforts of the National Cancer Institute’s Clinical Proteomic Tumor Analysis Consortium (CPTAC). For example, the proteomic data commons (https://proteomic.datacommons.cancer.gov/pdc/) provides the raw data resulting from the accumulation of knowledge with respect to cancer biology, which has significant secondary benefits. These raw data, which meet certain quality characteristics, allow users to test analysis pipelines. Raw data from clinical laboratories will also improve the transparency of the data analysis process, because processed data should correlate with disease processes across institutions, just like quantitative data in biofluid assays.

### Include Anatomic Pathologists in Assay Development and Data Interpretation

**Fig. 3:**
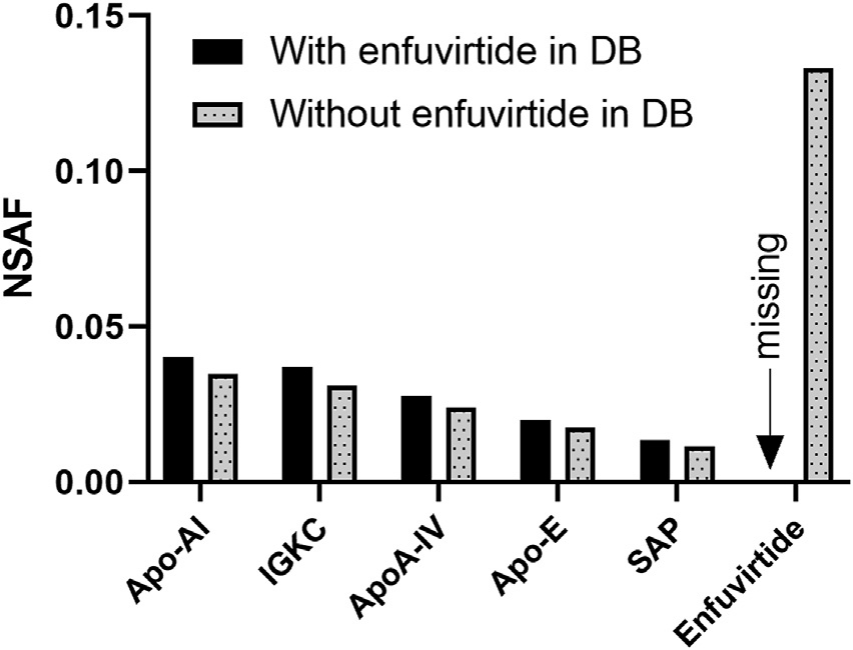
**An illustrated pitfall in amyloid typing.** Database searching was performed without and with the inclusion of an exogenous amyloidogenic substance, enfuvirtide (amino acid sequence YTSLIHSLIEESQNQQEKNEQELLELDKWASLWNWF). The data are from a clinical sample evaluated using the workflow described in reference (141). Protein abundance is expressed in the form of NSAF for the 5 most abundant amyloid-associated proteins in the sample in addition to enfuvirtide. Apo-AI, Apolipoprotein A-I; Apo-E, Apolipoprotein E; ApoA-IV, Apolipoprotein A-IV; DB, database; IGKC, Immunoglobulin kappa constant; SAP, Serum amyloid P-component.

As clinical mass spectrometry becomes increasingly sophisticated, there may be a desire to shift the entire operation to the hands of technical experts (*e.g.*, analytical chemists) or centralize testing in reference laboratories away from health care facilities. However, there is a significant potential for risk in the misinterpretation of proteomic data if results are not carefully interpreted in light of a patient’s clinical history and other laboratory testing results. There are also cases in which correct interpretation requires advanced or more obscure medical knowledge and a formal interface between anatomic pathologists and proteomic data can be essential. For example, Figure 3 illustrates a clinical case in which amyloid typing failed to identify the amyloid type (*i.e.*, the protein responsible for the amyloidosis), until the search database was modified to include an exogenous substance of clinical relevance to the patient.

## Conclusion

As a clinical diagnostic platform, IHC has deficiencies and we propose that solutions incorporating mass spectrometry, or more specifically, LC-MS/MS, have many advantages that will help advance diagnosis, prognosis, and therapeutic management based on tissue biopsies. Successful implementation over the long term will require careful attention to factors related to validation and quality monitoring. Because these methods differ fundamentally from other applications of LC-MS/MS in clinical laboratories, thoughtful approaches are needed for calibration, validation, and quality assurance and we look forward to new advances in this exciting direction for clinical proteomics.

## Conflict of interest

Dr Mandy Paulovich, M.D., Ph.D., is the founder of Precision Assays. The rest of the authors have nothing to disclose.
